# Betanin from Beetroot (*Beta vulgaris* L.) Regulates Lipid Metabolism and Promotes Fat Browning in 3T3-L1 Adipocytes

**DOI:** 10.3390/ph16121727

**Published:** 2023-12-14

**Authors:** Ho Seon Lee, Seung Min Choi, Sung Ho Lim, Chang-Ik Choi

**Affiliations:** Integrated Research Institute for Drug Development, College of Pharmacy, Dongguk University-Seoul, Goyang 10326, Republic of Korea; ghtjsrhtn@dongguk.edu (H.S.L.); tlsehdhs@dgu.ac.kr (S.M.C.); sho617@dgu.ac.kr (S.H.L.)

**Keywords:** 3T3-L1 cells, AMPK signaling pathway, beet, *Beta vulgaris*, betanin, fat browning

## Abstract

Fat browning, which converts white adipose tissue to brown, has attracted attention as a promising strategy for the treatment of obesity. Betanin (BT) has been reported to have potential anti-obesity activity. 3T3-L1 cells were differentiated for 7 days during BT treatment. The BT concentration range for the study was determined using an MTT assay, and lipid accumulation was evaluated by Oil-Red-O staining. The expression of protein level was analyzed by Western blot. Immunofluorescence images were performed with confocal microscopy to visually show the amount and location of thermogenesis factor uncoupling protein1 (UCP1) and mitochondria. qRT-PCR was performed to evaluate mRNA expression. BT inhibited lipid accumulation and increased the expression of UCP1, peroxisome-proliferator-activated receptor gamma (PPARγ), and PPARγ coactivator-1 alpha (PGC-1α). In addition, the increases in beige adipocyte-specific markers were observed, supporting BT-mediated browning of the fat tissue. The UCP1 was localized in the inner membrane of the mitochondria, and its expression was associated with mitochondrial activation. Consistent with this, the mRNA expression of mitochondrial biogenesis markers increased in 3T3-L1 cells after BT treatment. Immunofluorescence staining also indicated an increased number of mitochondria and UCP1, respectively. Moreover, BT inhibited lipogenesis and enhanced lipolysis and fatty acid oxidation. This mechanism has been suggested to be mediated by an adenosine monophosphate-activated protein kinase (AMPK) pathway. BT induces fat browning and regulates lipid metabolism via the AMPK-mediated pathway in 3T3-L1 cells, suggesting that BT can be a promising candidate for controlling obesity.

## 1. Introduction

Obesity is described as an abnormal fat accumulation, which is caused by an imbalance in the excessive intake of high-calorie foods and reduced energy consumption [1,2]. This leads to unhealthy weight gain, which is closely related to the development of various metabolic syndromes such as type 2 diabetes, dyslipidemia, cardiovascular disease, hypertension, osteoarthritis, and certain cancers [3]. Traditional treatments for obesity, such as diet restriction and exercise, are also available, but adjunctive pharmacotherapy may be recommended [4]. Some drugs used for obesity treatment carry the risk of serious side effects (i.e., potential abuse, heart disease, high blood pressure, stroke, and increased suicide rates), and several have been withdrawn from the market [5]. Therefore, the treatment of obesity using a natural compound, with a relatively low risk of side effects, is attracting attention [6,7]. Several recent studies have shown that natural compounds such as chrysin [8], thymol [9], trans-cinnamic acid [10], and resveratrol [11] can induce fat browning, which is considered a novel approach to the treatment of obesity [12,13].

White adipocytes store energy primarily in the form of triglycerides [14], while brown adipocytes generate heat, which increases energy expenditure [15]. Beige adipocytes, another type of adipocytes, have a similar function to brown adipocytes but are known to be derived from white adipocytes [16,17]. When white adipocytes exhibit the characteristics of brown adipocytes, it is called fat browning [18]. Several studies have shown that the energy-consuming properties of brown-like beige adipocytes could potentially be used in the treatment of obesity [19,20] by targeting thermogenesis, the main regulator of which is uncoupling protein1 (UCP1) [21]. This protein exists specifically in mitochondria and contributes to non-shivering thermogenesis, which consumes energy by generating heat instead of adenosine triphosphate (ATP) in the inner mitochondrial membrane [22]. Several factors that regulate the transcription of UCP1 include peroxisome-proliferator-activated receptor gamma coactivator-1 alpha (PGC-1α) and the PR domain containing 16 (PRDM16), all three of which are considered brown fat-specific markers. In addition, tumor necrosis factor receptor superfamily, member 9 (TNFRSF9, CD137), cell death inducing DFFA-like effector A (CIDEA), carboxy-terminal domain 1 (CITED1), fibroblast growth factor 21 (FGF21), T-box 1 (TBX1) and, transmembrane protein 26 (TMEM26) are beige-fat specific markers, indicating that fat browning is promoted as the corresponding marker increased [23].

**Figure 1:**
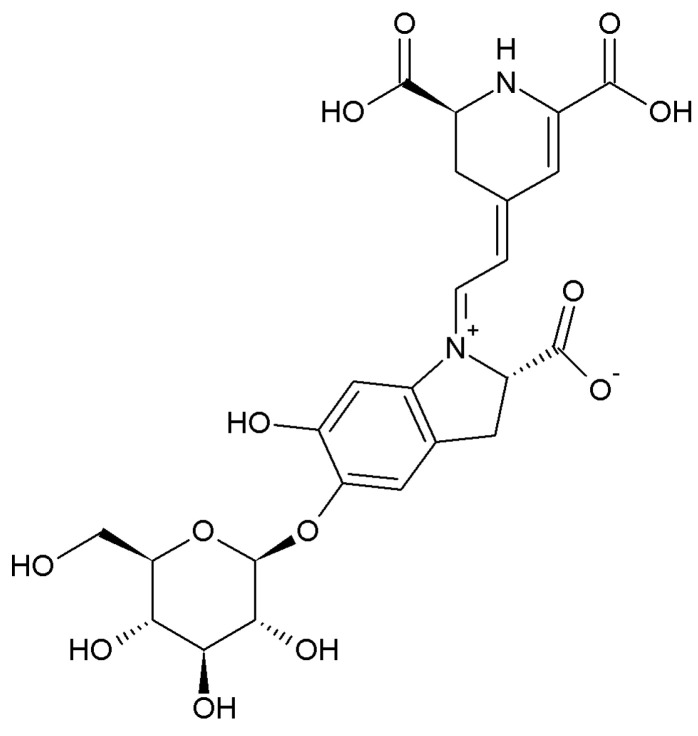
Chemical structure of betanin (BT).

*Beta vulgaris* Linnaeus (called beetroot) is a well-known functional health food and is widely cultivated and consumed around the world. These beetroots are known to be rich in plant compounds such as ascorbic acid, carotenoids, phenolic acids, flavonoids, and betalains [24]. Betanin (BT; betanidin 5-*O*-β-glucoside; Figure 1) belongs to the betalain family and is known to play an antioxidant role with excellent electron donor functions of phenolic and cyclic amine groups [25]. In addition, BT exerts various physiological activities such as antioxidant, antihypertensive, anti-inflammatory, anticancer, and antidiabetic activities [26]. Furthermore, a lipid-lowering effect has been confirmed in the aqueous extract of beetroot [27]. However, research on the effect of BT in beets on fat browning is currently lacking.

Here, we investigated the effect of betanin on the protein and mRNA levels of several pathways involved in fat browning in 3T3-L1 white adipocytes. In addition, the mechanism related to the regulation of thermogenic proteins mediated by the AMPK signaling pathway was studied. This study presents the effect of BT on brown adiposity and provides the potential of beetroot-derived substances as anti-obesity agents.

## 2. Results

### 2.1. Effects of BT on Cell Viability and Lipid Accumulation in 3T3-L1 Adipocytes

**Figure 2:**
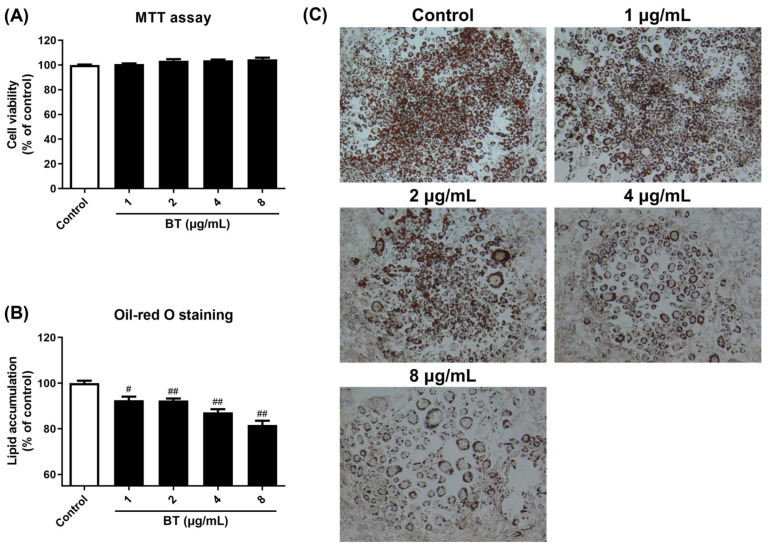
BT inhibits lipid accumulation in 3T3-L1 adipocytes. Cell viability of 3T3-L1 pre-adipocytes (**A**). The cells were treated with BT and incubated for 48 h. Lipid droplets stained with Oil-Red-O solution were extracted with isopropanol to evaluate lipid accumulation (**B**). Representative images of Oil-Red-O staining of 3T3-L1 cells taken at 100× magnification (**C**). The cells on day 7 of the differentiation process were stained with Oil-Red-O. Data are presented as mean ± SEM (n = 3). # *p* < 0.05 and ## *p* < 0.01 compared with the control.

To determine the influence of BT on cell viability, we performed an MTT assay in 3T3-L1 cells. Cells were treated with BT at concentrations of 1–8 μg/mL for 48 h. BT had no effect on cell viability at concentrations of up to 8 μg/mL (Figure 2A). As the development of obesity involves adipogenesis and lipid accumulation, we investigated the inhibitory effects of BT on lipid accumulation using Oil-Red-O staining. BT reduced lipid accumulation in a concentration-dependent manner compared to the control cells (Figure 2B). Furthermore, micrographs confirmed that BT treatment reduced the size of lipid droplets and increased the number of small lipid droplets (Figure 2C).

### 2.2. Effects of BT on Browning Factors in 3T3-L1 Adipocytes

**Figure 3:**
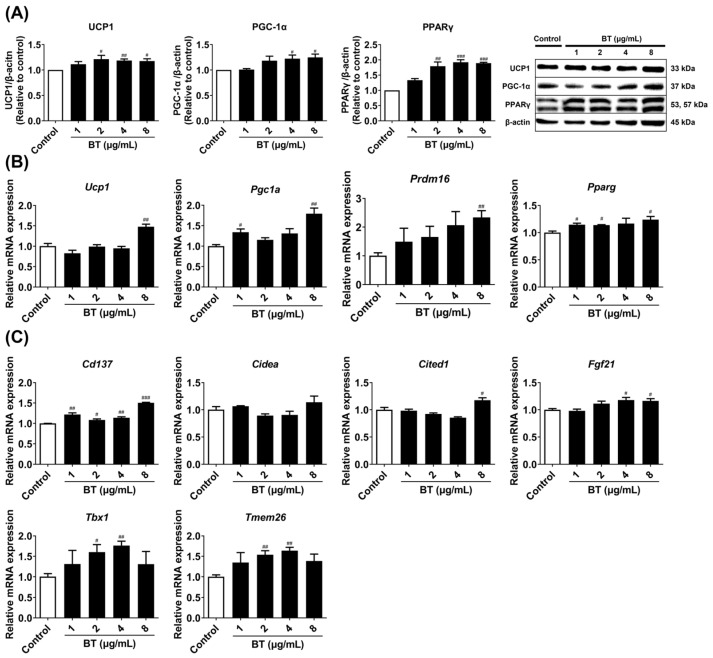
BT induces browning in 3T3-L1 adipocytes. BT elevates thermogenic factors at the protein (**A**) and mRNA levels (**B**). Beige-specific genes (**C**). Each was analyzed by Western blot and qRT-PCR. β-actin was used as a loading control. Target gene mRNA levels were normalized to GAPDH using the 2^−∆∆^ Ct method. Data are presented as mean ± SEM (n = 3). # *p* < 0.05, ## *p* < 0.01, and ### *p* < 0.001 compared with the control.

A concentration range of 1–8 µg/mL was used to determine whether BT induced the expression of browning markers in 3T3-L1 white adipocytes. Treatment with BT enhanced all thermogenic factors. The protein expression of UCP1, PGC-1α, and PPARγ was increased by treatment with BT (Figure 3A). Furthermore, the mRNA expression of the corresponding genes *Ucp1*, *Pgc1a*, *Pparg*, and *Prdm16* also showed similar trends (Figure 3B). Beige-specific genes are specifically expressed in brown-like adipocytes and indicate fat browning. BT treatment improved the expression of all of these genes (such as *Cd137*, *Cidea*, *Cited1*, *Fgf21*, *Tbx1*, and *Tmem26*). These results suggest that BT has the potential to promote fat browning by inducing thermogenic markers in 3T3-L1 adipocytes.

### 2.3. Effects of BT on Mitochondrial and UCP1 Activation in 3T3-L1 Adipocytes

**Figure 4:**
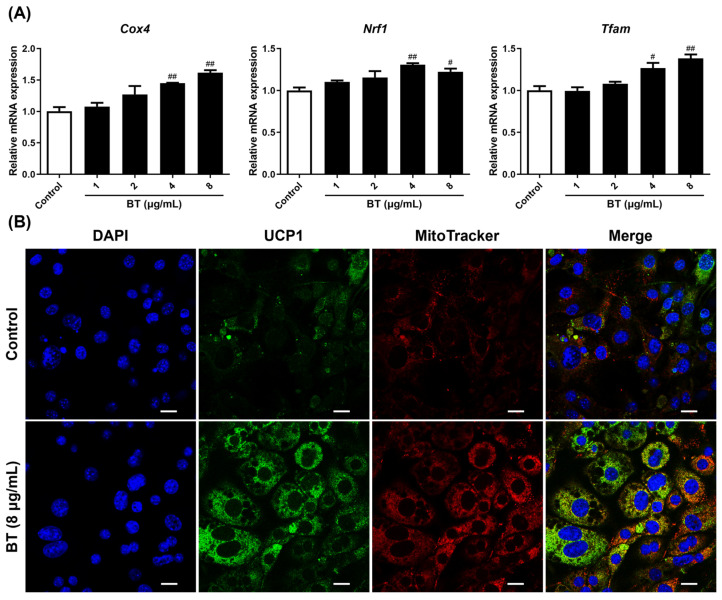
BT increases UCP1 and mitochondria in 3T3-L1 adipocytes. Mitochondrial biogenesis markers (**A**). The effect of BT on mitochondrial biogenesis genes was confirmed by qRT-PCR analysis. Target gene mRNA levels were normalized to GAPDH using the 2^−∆∆^ Ct method. Immunofluorescent staining (**B**). The cells were treated with 8 µg/mL of BT for 7 days of differentiation for immunofluorescent staining. Representative images were each captured at 60× magnification, and scale bars represent 20 µm. UCP1-FITC was applied for immunofluorescence and stained with MitoTracker Red and DAPI. Data are presented as mean ± SEM (n = 3). # *p* < 0.05 and ## *p* < 0.01 compared with the control.

UCP1 is a mitochondrial-specific protein and is known to play an important role in thermogenesis. PGC-1α is also known to affect mitochondrial biogenesis. We already confirmed that BT increased protein and mRNA levels of UCP1 and PGC-1α (Figure 3A,B). We then measured the mRNA expression of mitochondrial biogenesis markers (*Cox4*, *Tfam*, *Nrf1*). BT increased the expression of *Cox4* and *Tfam* mRNA concentration-dependently, and the increase in *Nrf1* expression was highest at a BT concentration of 4 μg/mL (Figure 4A). Furthermore, treatment with BT enhanced UCP1 levels and the number of mitochondria compared to control cells (Figure 4B). Thus, BT affects fat browning by activating mitochondria and UCP1.

### 2.4. Effects of BT on Lipid Metabolism in 3T3-L1 Adipocytes

**Figure 5:**
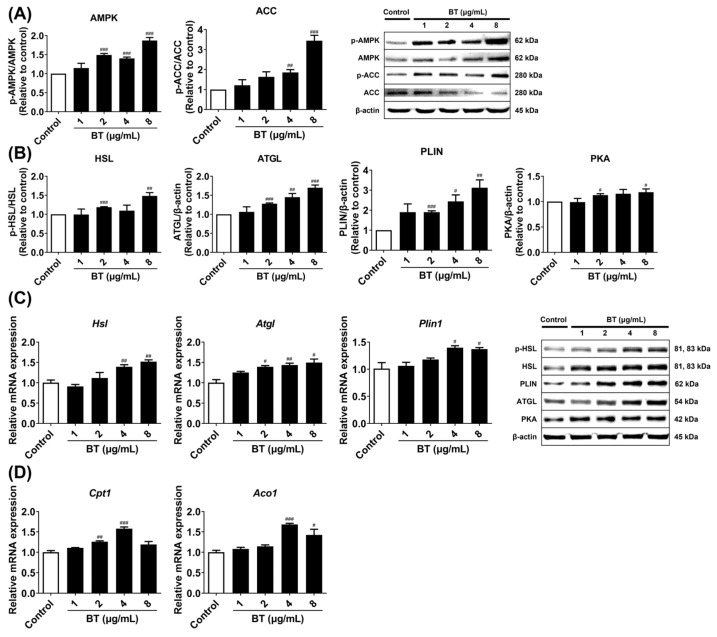
BT regulates lipid metabolism in 3T3-L1 adipocytes. Lipogenesis proteins (**A**). Lipolysis proteins (**B**) and mRNAs (**C**). Fatty acid oxidation genes (**D**). Each was analyzed by Western blot and qRT-PCR. β-actin was used as a loading control. Target gene mRNA levels were normalized to GAPDH using the 2^−∆∆^ Ct method. Data are presented as mean ± SEM (n = 3). # *p* < 0.05, ## *p* < 0.01, and ### *p* < 0.001 compared with the control.

We measured the protein levels of AMPK and ACC, two factors associated with lipogenesis, via Western blotting analysis, and analyzed the mRNA expression of *Aco1* and *Cpt1*, two genes involved in fatty acid oxidation, using qRT-PCR. BT significantly increased p-AMPK/AMPK and p-ACC/ACC ratios (Figure 5A). In addition, the expression of *Aco1* and *Cpt1*, which are genes associated with fatty acid oxidation, were elevated, but tended to decrease at the highest concentration (Figure 5D). Furthermore, the levels of HSL, ATGL PLIN, and PKA proteins related to lipolysis were evaluated by Western blotting analysis, whereas the mRNA expression of *Hsl*, *Atgl,* and *Plin1* was investigated using qRT-PCR. BT treatment decreased the size but increased the number of lipid droplets (Figure 2B,C). BT activated all lipolysis markers, especially ATGL, PLIN, PKA, *Hsl*, and *Atgl* showed a concentration-dependent increase in expression (Figure 5B,C). These results suggested that BT contributed to the maintenance of fat browning by regulating lipid metabolism.

### 2.5. Effects of BT on UCP1 Expression via Activation of the AMPK Signaling Pathway in 3T3-L1 Adipocytes

**Figure 6:**
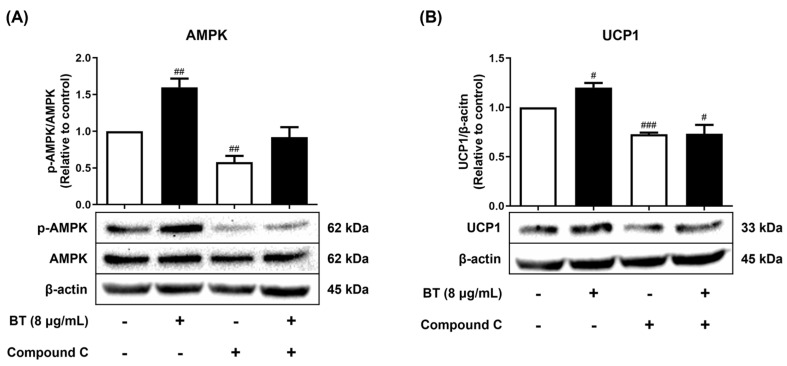
BT induces fat browning via the AMPK pathway in 3T3-L1 adipocytes. The protein levels of AMPK (**A**) and UCP1 (**B**) were analyzed using Western blot. BT was treated at a concentration of 8 µg/mL. The cells were pretreated with Compound C (10 μM) for 2 h followed by BT treatment and harvested 7 days after differentiation. β-actin was used as a loading control. Data are presented as mean ± SEM (n = 3). # *p* < 0.05, ## *p* < 0.01, and ### *p* < 0.001 compared with the control.

BT affected fat browning by increasing UCP1 protein and mRNA levels (Figure 3A,B). The AMPK pathway is well known as a pathway involved in fat browning, and BT also contributed to AMPK activation (Figure 5A). We showed that BT promotes fat browning by regulating the UCP1 protein levels via the AMPK signaling pathway. BT clearly activated AMPK in 3T3-L1 adipocytes, and activity was reduced when cells were exposed to compound C, an AMPK inhibitor. Additionally, when cells were exposed to the inhibitor and BT sequentially, the expression that was decreased by the inhibitor was restored (Figure 6A). Furthermore, this trend was also observed for UCP1 protein under BT and inhibitor treatment conditions (Figure 6B). Consequently, our data suggested that BT has the potential to regulate the expression of UCP1 by mediating the AMPK pathway. Taken together, all results suggest that AMPK signaling is potentially involved in BT-induced fat browning in 3T3-L1 adipocytes.

## 3. Discussion

Obesity is becoming increasingly common worldwide, with 18% of men and 21% of women expected to become obese by 2025 [28]. Obesity is a risk factor that can cause various diseases, and various attempts have been made to treat it. Fat browning is an approach to improving obesity that has drawn attention and involves beige adipocytes, which are a transdifferentiated form of white adipocytes having functions similar to those of brown adipocytes [29]. Beetroot and BT have been confirmed to have many bioactive effects via many studies. Various pharmacological activities of BT have been reported, and lipid-lowering effects have also been confirmed in the aqueous extract of beetroot. Although many studies investigating beetroot and BT have been conducted, studies on energy homeostasis due to fat browning and its anti-obesity activity are currently lacking, and the associated signaling pathways are also unclear. The present study aimed to elucidate the potential signaling pathways related to lipid metabolism and fat browning properties of BT in beetroot.

The activity of UCP1, a protein in the mitochondrial inner membrane of adipocytes, is the key to the mechanism of treatment for fat browning-induced obesity [30]. UCP1 expression is extensively controlled at the transcriptional level, with various variables implicated in regulating the activity of browning-related factors. The activation of β3-adrenergic receptors (β3-ARs) has an effect on UCP1 production [31]. Its activation causes an interaction that stimulates cyclic AMP (cAMP) and PKA [32], which triggers p38 mitogen-activated protein kinase (MAPK) phosphorylation and activation, and in turn, stimulates PGC-1α and activates the transcription factor 2 (ATF-2), which is directly implicated in regulating UCP1 expression [33]. Furthermore, co-activators PGC-1α and PRDM16 form complexes with transcription factors such as PPARγ, resulting in UCP1 transcription and browning [34]. In our study, BT treatment increased the mRNA expression of *Pgc1a*, *Pparg*, *Prdm16*, and *Ucp1* and also increased the expression of PGC-1α, PPARγ, and UCP1 at the protein level. These markers are related to thermogenesis in adipose tissue, and as they increase, it can be assumed that energy consumption and fat browning are activated. Additionally, BT increased the expression of beige adipose-specific markers *Cd137*, *Cidea*, *Cited1*, *Fgf21*, *Tbx1*, and *Tmem26*. These targets are markers identified when white adipocytes are browned and induced to brown-like adipocytes, which are called beige adipocytes, and their increase is also an indicator of the activation of fat browning.

PPARγ is known to encourage adipocyte development as a master adipogenic transcription factor [35]. Therefore, the reduction in PPARγ is associated with the suppression of adipogenesis, which is considered an anti-obesity effect [36]. Conversely, in some cases, an increase in PPARγ is interpreted positively for thermogenesis and fat browning. An increase in PPARγ has been confirmed to be a factor that contributes to increased fat browning via interaction with other thermogenic factors [37]. In particular, PPARγ is involved in the transcription of UCP1 and forms a complex with PGC-1α and PRDM16 [38]. In addition, agonists of PPARγ contribute to browning by stabilizing the PRDM16 protein [39]. In this study, treatment with BT slightly increases PPARγ, which, along with increases in other thermogenic markers, UCP1, PGC-1α, and PRDM16, suggests that they contribute to fat browning.

AMPK activation is a regulator of cellular metabolism and phosphorylation of metabolic protein substrates and is involved in adipose tissue development, thermogenesis, mitochondrial biogenesis, and fatty acid oxidation [40,41]. Phosphorylated and activated AMPK phosphorylates ACC and converts it to an inactive state, which reduces the level of malonyl-CoA and thus restores the activity of CPT1, resulting in the suppression of lipogenesis and promotion of fatty acid oxidation [42]. Furthermore, AMPK activity positively regulated PGC-1α expression and mitochondria levels [42]. The ratios of p-AMPK/AMPK and p-ACC/ACC were increased in our study. On the other hand, BT increased PGC-1α in a dose-dependent manner along with AMPK, and mitochondrial biogenesis markers (*Cox4*, *Nrf1*, and *Tfam*) were also promoted as a follow-up reaction. These findings were also supported by the immunofluorescence results. Additionally, PGC-1α is a key factor regulating mitochondrial biogenesis and induces the expression of several mitochondrial genes, including *Nrf1*/*2* and *Tfam* [43]. *Nrf1*/*2* activation could potentially have an influence on obesity because it is implicated in inflammation via antioxidant activity [44,45]. *Aco1* and *Cpt1*, which are targets involved in β-oxidation, were also enhanced, contributing to UCP1 activation. BT induced the activation of UCP1 and mitochondria. Furthermore, BT stimulated AMPK, an important factor in lipid metabolism, which indicated that it had a significant influence on fat browning.

The activation of β3-ARs in brown adipocytes stimulates cAMP and PKA, leading to phosphorylation of HSL to increase lipolysis [46]. Another enzyme involved in lipolysis is ATGL. This enzyme is involved in the hydrolysis of triglycerides (TG) to diglycerides, while HSL prefers diglycerides as a substrate and has a broad substrate specificity [47]. In addition, when PKA is activated, it induces the phosphorylation of PLIN, which assists lipase in accessing lipid droplets. Phosphorylated PLIN removes the protective effect of lipid droplets, thus promoting lipolysis [48]. The free fatty acid released from lipolysis activates UCP1 as an agonist. *Cpt1* increases the oxidation of *Aco1*, allowing it to increase free fatty acid transport into mitochondria [49], which serves as fuel to sustain fatty acid oxidation and thermogenesis [50]. Our results confirmed that BT treatment increased ATGL, HSL, PKA, and PLIN, which are related to lipolysis, and *Cpt1* and *Aco1*, which are related to fatty acid oxidation. This indicates that BT contributes to fatty acid oxidation, and it is possible that it regulates UCP1 expression and promotes fat browning.

We investigated the AMPK-mediated pathway to determine its involvement in the BT-induced increase in UCP1. Compound C was used, which is a well-known AMPK inhibitor. Treatment with compound C reduced the expression of UCP1, which was increased by BT treatment, along with inhibition of AMPK. This suggested that the thermogenesis-enhancing activity of BT was influenced by the AMPK-mediated pathway. Different pathways other than AMPK-mediated pathways, such as β3-ARs, MAPK, and PKA, are also involved in promoting fat browning [51]. There may still be associations other than the betanin and AMPK pathways, and further research is needed. Nonetheless, our study only evaluated the AMPK-mediated pathway among many mediating pathways related to fat browning. In addition, only compound C, an inhibitor of AMPK, was used in this study. The increase in thermogenesis by BT may be increased by other pathways. Thus, the identification of other mediating pathways via further study could enhance our understanding of the fat browning effects of BT.

## 4. Materials and Methods

### 4.1. Reagents

Mouse 3T3-L1 pre-adipocytes were provided by the Korean Cell Line Bank (Seoul, Republic of Korea). BT, dexamethasone (DEX), insulin, phosphate-buffered saline (PBS), 10% formalin, Oil-Red-O, and compound C (dorsomorphin) were obtained from Sigma-Aldrich (St. Louise, MO, USA). 3-isobutyl-1-methylxanthine (IBMX), goat anti-rabbit IgG secondary antibody, goat anti-mouse IgG secondary antibody, and isopropanol were obtained from Merck (Union County, NJ, USA). Newborn bovine calf serum (NCS), fetal bovine serum (FBS), Dulbecco’s modified eagle’s medium/high glucose (DMEM), penicillin-streptomycin solution (P-S), and 4′,6-diamidino-2-phenylindole (DAPI) were obtained from Thermo Fisher Scientific (Waltham, MA, USA). Thiazolyl blue tetrazolium bromide (MTT) and dimethyl sulfoxide (DMSO) were purchased from Glentham Life Sciences (Corsham, UK). MitoTracker^®^ Red CMXRos was obtained from Cell Signaling Technology (Danvers, MA, USA). Antibodies against proliferator-activated receptor gamma (PPARγ), phospho-adenosine monophosphate-activated protein kinase alpha (AMPK) α, AMPKα, phospho-acetyl-CoA carboxylase (ACC), ACC, phospho-hormone-sensitive lipase (HSL), HSL, adipose triglyceride lipase (ATGL), perilipin (PLIN), protein kinase A (PKA), and β-actin were obtained from Cell Signaling Technology. UCP1, UCP1-Fluorescein isothiocyanate (FITC), and PGC-1α were purchased from Santa Cruz Biotechnology (Santa Cruz, CA, USA). iQ™ SYBR^®^ Green Supermix was purchased from BIO-RAD (Hercules, CA, USA). NucleoZOL and the NucleoSpin^®^ RNA Set for NucleoZOL were obtained from Macherey-Nagel (Düren, Germany). ReverTra Ace^®^ qPCR RT Kit was obtained from TOYOBO (Osaka, Japan). Triton^®^ X-100 was obtained from Promega (Madison, WI, USA).

### 4.2. Cell Culture and Differentiation

Differentiation of 3T3-L1 cells proceeded according to the method we established [13]. 3T3-L1 pre-adipocytes were grown in DMEM with 10% NCS and 1% P-S supplements for 4 days at 37 °C in a 5% CO_2_ incubator. Every 2 days, the culture media was replaced. For 3 days, sufficiently confluent cells were cultured in a differentiation-inducing medium. This medium contained 10% FBS, 10 μg/mL insulin, 1 μM DEX, and 0.5 mM IBMX in DMEM. Subsequently, the cells were incubated for 2 days in a maturation medium consisting of DMEM with 10% FBS and 10 μg/mL insulin. Every 2 days, the maturation medium was changed. During treatments, cells were maintained in a complete medium containing BT (1–8 μg/mL, dissolved in DMSO) for 7 days prior to further analysis. The mechanisms involved in browning were investigated by pretreatment of 3T3-L1 cells with the selective AMPK inhibitor, compound C (dorsomorphin; 10 μM), together with differentiation medium and maturation medium for 2 h, respectively, until the cells were harvested.

### 4.3. MTT Assay

Cell viability of BT on 3T3-L1 cells was determined using the MTT assay [7]. Pre-adipocytes (1 × 10^4^ cells/well) were seeded in a 96-well plate. After incubation for 24 h, cells were treated with BT (1–8 μg/mL) for 48 h. MTT (5 mg/mL) solution diluted with PBS was added. The plate was then incubated at 37 °C for 2 h with 5% CO_2_. The formazan crystals that were formed were dissolved in DMSO after the supernatant was removed. Cell viability was confirmed by absorbance measurement at 540 nm using an xMark™ Microplate Absorbance Spectrophotometer (BIO-RAD).

### 4.4. Oil-Red-O Staining

Oil-Red-O staining was performed as described in our previous article [13]. Briefly, pre-adipocytes (1 × 10^5^ cells/well) were seeded in 24-well plates. After 7 days of differentiation, the cell layer was washed twice with PBS. Next, 10% formalin (*v*/*v*) was applied, and the cells were fixed for 30 min at room temperature and washed with deionized water (DW). Each well was then filled with a 0.5% Oil-Red-O dye solution in isopropanol, and the cells were incubated at room temperature for 30 min. The cells were then washed with DW, observed under a microscope, and photographed. The plate was then incubated at room temperature overnight until the DW completely dried out. The dyeing solution was extracted by adding isopropanol to each well, and the plate was shaken for 30 min using a vortex mixer. After transferring to a new 96-well plate, the absorbance was measured at 520 nm using an xMark™ Microplate Absorbance Spectrophotometer (BIO-RAD) to confirm lipid accumulation.

### 4.5. Mitochondrial Analysis and Immunofluorescence

The pre-adipocytes were cultured and differentiated in 24-well plates (1 × 10^5^ cells/well) on which sterilized coverslips were placed as described in our previous article [13]. After differentiation for 7 days, MitoTracker^®^ Red CMXRos (50 nM) for mitochondrial staining was added to the growth medium in mature adipocytes, and cells were incubated at 37 °C in an incubator with 5% CO_2_ for 30 min. The cells were then washed three times with PBS and fixed at room temperature for 15 min with 10% formalin. After fixation, the immunofluorescence staining was performed. The cells were washed three times with PBS and treated with a blocking buffer (5% bovine serum albumin, 0.1% Triton^®^ X-100 in PBS) for 1 h at 4 °C. Subsequently, the cells were washed with blocking buffer, and the FITC-conjugated primary antibody (UCP1, dilution 1:250) was added. The cells were incubated overnight at 4 °C, followed by three washes with PBS for 5 min. For staining of nuclei, cells were treated for 1 min with DAPI (dilution 1:1000) and then washed with PBS. Fluorescence images were captured using a Nikon C1 confocal laser scanning microscopy instrument with EZ-C1 software 3.9 (Nikon, Tokyo, Japan).

### 4.6. Western Blotting Analysis

The preadipocytes were seeded in 6-well plates at 1 × 10^6^ cells/well as described earlier [7]. After 7 days, the differentiated 3T3-L1 adipocytes were scraped with ice-cold PBS. The cells were centrifuged (14,000 rpm, 5 min), and the supernatant was removed. Next, 100 μL of lysis buffer was added to release the pellet, which was incubated for 30 min on ice. The supernatant was centrifuged (14,000 rpm, 25 min) and transferred to a new 1.5 mL tube. The protein volume was quantified to 40 µg and filled with sample buffer (mix 900 μL of 4× Laemmli sample buffer and 100 μL of β-ME) to obtain a total volume of 20 μL. After 10 min of incubation at 100 °C using a heating block, the cells were centrifuged (14,000 rpm, 3 min). Furthermore, 10% separating gel was prepared, proteins were loaded, and the gel was electrophoresed for 1 h using a PowerPac (BIO-RAD). The immun-Blot^®^ PVDF membrane (BIO-RAD) was used to transfer proteins for 1 h. The membrane was then blocked for 1 h in a solution of 5% skim milk in Tris-buffered saline and 0.1% Tween 20 (TBS-T). After washing with TBS-T, incubating at 4 °C with a primary antibody (diluted 1:1000, containing 5% bovine serum albumin in TBS-T), the membrane was washed with TBS-T and incubated for 1 h with anti-rabbit or mouse IgG secondary antibody (diluted 1:4000, containing 5% skim milk in TBS-T). Protein signals were visualized using Chemi-Doc (BIO-RAD). Band intensities were quantified using Image Lab software 3.0 (BIO-RAD).

### 4.7. Quantitative Real-Time Polymerase Chain Reaction (qRT-PCR)

Adipocytes differentiated in 6-well plates (5 × 10^6^ cells/well) were scraped with ice-cold PBS and centrifuged (14,000 rpm, 5 min, 20 °C), and the supernatant was removed. Subsequently, the cells were dissolved in 1 mL of NucleoZOL, and a total RNA isolation kit (NucleoSpin^®^ RNA Set for NucleoZOL, Macherey-Nagel) was used. Total RNA (1 μg) was converted to cDNA using the ReverTra Ace^®^ qPCR RT Kit (TOYOBO). qRT-PCR was performed on the CFX384 Touch Real-Time PCR Detection System (Bio-Rad) using the iQ™ SYBR Green Supermix. The primer sequences used in this study are listed in Table 1.

### 4.8. Statistical Analysis

All data are expressed as the mean ± standard error of the mean (SEM). Student’s *t*-test was used to determine the difference between means. Statistical significance between each sample is considered at *p* < 0.05.

## 5. Conclusions

BT induces fat browning by increasing the expression of thermogenesis-related biomarkers and supports browning by increasing the beige fat-specific markers. In addition, BT provides the fuel necessary for thermogenesis by increasing fatty acid oxidation and lipolysis, and we show the possibility that BT-induced thermogenesis is mediated by the AMPK pathway. Overall, BT induced fat browning by enhancing markers related to energy expenditure and lipid metabolism, which suggests that this could represent a promising strategy for controlling obesity.

## Figures and Tables

**Table 1:** The primer sequence used for qRT-PCR.

Gene	Forward	Reverse
*Aco1*	ATCCAGACTTCCAACATFAG	AACCACATGATTTCTTCAGG
*Atgl*	TTCACCATCCGCTTGTTGGAG	AGATGGTCACCCAATTTCCTC
*Cd137*	GGTCTGTGCTTAAGACCGGG	TCTTAATAGCTGGTCCTCCCTC
*Cidea*	CGGGAATAGCCAGAGTCACC	TGTGCATCGGATGTCGTAGG
*Cited1*	AACCTTGGAGTGAAGGATCGC	GTAGGAGAGCCTATTGGAGATGT
*Cox4*	TGACGGCCTTGGACGG	CGATCAGCGTAAGTGGGGA
*Cpt1*	GTGTTGGAGGTGACAGACTT	CACTTTCTCTTTCCACAAGG
*Fgf21*	CGTCTGCCTCAGAAGGACTC	TCTACCATGCTCAGGGGGTC
*Hsl*	TGTCGTAGTGGCCGTTCTGA	CACACTGAGGCCTGTCTCGTT
*Nrf1*	GCTAATGGCCTGGTCCAGAT	CTGCGCTGTCCGATATCCTG
*Pgc1a*	ATGTGCAGCCAAGACTCTGTA	CGCTACACCACTTCAATCCAC
*Plin1*	GCAAGAAGAGCTGAGCAGAC	AATCTGCCCACGAGAAAGGA
*Pparg*	CAAGAATACCAAAGTGCGATCAA	GAGCTGGGTCTTTTCAGAATAATAAG
*Prdm16*	GATGGGAGATGCTGACGGAT	TGATCTGACACATGGCGAGG
*Tbx1*	AGCGAGGCGGAAGGGA	CCTGGTGACTGTGCTGAAGT
*Tfam*	ATGTGGAGCGTGCTAAAAGC	GGATAGCTACCCATGCTGGAA
*Tmem26*	CCATGGAAACCAGTATTGCAGC	ATTGGTGGCTCTGTGGGATG
*Ucp1*	CCTGCCTCTCTCGGAAACAA	GTAGCGGGGTTTGATCCCAT
*GAPDH*	TTGTTGCCATCAACGACCCC	GCCGTTGAATTTGCCGTGAG

## Data Availability

The data presented in this study are available on request from the corresponding author.
